# Translation and cross-cultural adaptation of Integrated Palliative Care Outcome Scale for Dementia

**DOI:** 10.1177/02692163251347826

**Published:** 2025-07-13

**Authors:** Linghui Chen, Katherine E Sleeman, Huichan Huang, Yihan Mo, Andy Bradshaw, Clare Ellis-Smith

**Affiliations:** 1 Cicely Saunders Institute of Palliative Care, Policy and Rehabilitation, Florence Nightingale Faculty of Nursing, Midwifery and Palliative Care, King’s College London, London, UK; 2 Department of Geriatric Medicine, Fuzhou University Affiliated Provincial Hospital, Fuzhou, China

**Keywords:** Outcome and process assessment, palliative care, dementia, qualitative research, cross-cultural adaptation

## Abstract

**Background:**

The Integrated Palliative Care Outcome Scale for Dementia (IPOS-Dem) was developed to assess symptoms and concerns comprehensively for people with dementia. There is a high demand for comprehensive assessment of people with dementia in China.

**Aim:**

To translate and culturally adapt the IPOS-Dem into Chinese.

**Design:**

Conceptual equivalence, forward and backward translations, and expert review were performed to develop a prototype Chinese version. Two rounds of cognitive interviews were conducted to ensure the items and scoring format were clearly expressed in the Chinese version.

**Setting/participants:**

Professionals, including a physician, a nurse, a linguistic researcher and a humanities researcher, were involved in the prototype Chinese version development. A purposive sample of 12 health care professionals working in three Chinese nursing homes participated in the cognitive interviewing.

**Results:**

The Chinese version was perceived as clinically useful. Challenges arose regarding comprehension of some items due to difficulties in translating the precise meanings. These included ‘Drowsiness (sleepiness)’, ‘Difficulty communicating’ and ‘Do you think s/he felt at peace?’. Considering how a symptom affects an individual presented was also challenging for respondents, as they needed to judge whether the symptom was present and/or causing distress. Selecting the appropriate term to name the measure elucidated the current understanding of dementia and palliative care in China, both of which remain poorly understood.

**Conclusion:**

This study highlighted the importance of cultural adaptation in conveying meanings across cultures. Most items were translatable and conceptually equivalent. The term ‘at peace’ and the concept of ‘being affected’ generated the most challenges in comprehension and judgement.

**What is already known about the topic?**

- People with dementia may experience a high symptom burden due to dementia and multiple co-morbidities.
- The Integrated Palliative Care Outcome Scale for Dementia (IPOS-Dem) is a person-centred outcome measure to support comprehensive assessment of symptoms and concerns experienced by people with dementia.
- Taking cultural and local contexts into account is essential when incorporating person-centred outcomes across different settings.

**What this paper adds?**

- We introduce a cultural adaptation of the IPOS-Dem, integrating nuances related to the Chinese language and cultural traits.
- This paper underscores the significance of cultural adaptation in elucidating, reformulating, and effectively conveying meanings and concepts across diverse cultures.
- The study identifies challenges linked with person-centred outcome measurement for people with dementia in China.

**Implications for practice**

- Consistent refinement, ongoing education and essential training are vital for improving the use of IPOS-Dem.
- The Chinese IPOS-Dem is ready for further psychometric testing to enhance its validity, reliability, feasibility and acceptability for clinical use in nursing home settings.
- Integrating IPOS-Dem into dementia care settings could support person-centred and holistic care, which will require a shared understanding of terms, concepts and philosophies of care among involved parties.

## Background

Dementia is a syndrome, usually chronic and progressive, that results in the deterioration of cognitive function (i.e. the ability to process thought) beyond what would be expected from the usual consequences of biological ageing.^1,2^ Globally, it is estimated that more than 55 million people have dementia, over 60% of whom live in low- and middle-income countries. In China, it is estimated that 15.07 million adults aged 60 years or older live with dementia, accounting for a quarter of people with dementia worldwide.^3^

People with dementia may experience a high symptom burden due to dementia and multiple chronic long-term conditions, resulting in a negative impact on quality of life.^4,5^ However, people with dementia tend to die in residential care, in acute hospitals or at home with limited access to palliative care.^6,7^ Early identification of palliative care needs and concerns through systematic comprehensive assessment has the potential to benefit individuals. Nonetheless, since people with dementia who are verbally compromised may be unable to express whether they are feeling a symptom, it is challenging to identify symptoms and concerns comprehensively in people with dementia,^4^ leading to distress. To fill this gap, the Integrated Palliative Care Outcome Scale for Dementia (IPOS-Dem) was developed as a person-centred outcome measure.

IPOS-Dem is derived from the Palliative care Outcome Scale (POS) and Integrated Palliative care Outcome Scale (IPOS).^8,9^ IPOS-Dem is a proxy-reported outcome measure allowing comprehensive assessment of symptoms and concerns experienced by people with dementia.^10^ It is a brief questionnaire, which is designed to be used by staff who provide direct care in care homes. The extent of common symptoms and problems to which people with dementia are affected is assessed and scored.^10^ IPOS-Dem has been demonstrated to be a feasible and acceptable tool for implementation in routine care in the residential care homes in the United Kingdom.^4^ It has been adapted to Swedish,^11^ German^5^ and Swiss German.^12^ Different versions are freely available at https://pos-pal.org/.

There is a lack of a comprehensive person-centred outcome measure for people with dementia in China. Therefore, the aim of this study is to translate and culturally adapt IPOS-Dem into Chinese and to develop a Chinese version of the questionnaire as a foundation for psychometric testing.

## Methods

**Figure 1.**
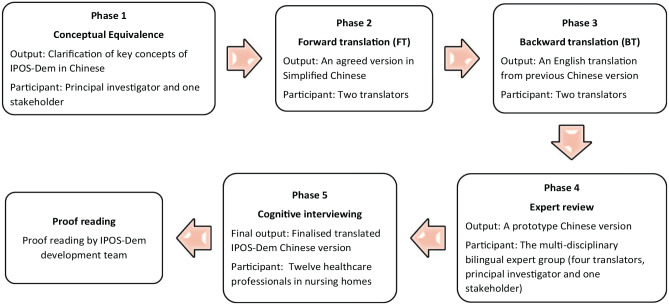
The process of translation and cross-cultural adaption (adapted from POS Family of Measures Manual for Translation, Cross-Cultural Adaptation and Psychometric Testing^13^).

The translation and cognitive interviewing were conducted according to the best practice guidance of the Palliative care Outcome Scale (POS) family of measures for translation and cross-cultural adaptation.^13^ Figure 1 gives an overview of the different phases, which were conducted from June 2023 to May 2024.

### Phase 1: Conceptual equivalence

Conceptual equivalence ensures that ideas have the same meaning across different cultures or languages. An item-by-item review was conducted by LC and one healthcare professional working in a Chinese nursing home to identify (1) the fundamental concepts for understanding the scope and objectives of IPOS-Dem or (2) items that may be subject to varying interpretations. Then, informal discussions with three healthcare professionals with expertise in caring for older people were used to explore the potential Chinese equivalent definitions for those concepts and items.

### Phase 2: Forward translation

Two bilingual translators independently completed two forward translations from English to Simplified Chinese. These two translations (FT1, FT2) were further scrutinised by a research team member and any discrepancies were discussed with the two translators to form forward translation three (FT3: an agreed version in Simplified Chinese).

### Phase 3: Backward translation

Two backward translations (BT1, BT2) were completed by two independent bilingual translators blinded to the original English version.

### Phase 4: Expert review

The multi-disciplinary bilingual expert group included a member from the research team, one health care professional familiar with IPOS-Dem and all four translators. The group synthesised and consolidated the backward translations into backward translation three (BT3: an English translation from the previous Chinese version). The expert group compared, evaluated, revised and consolidated items and response format between BT3 and initial IPOS-Dem items, which aimed to develop the prototype Chinese version ready for cognitive interviewing.

### Phase 5: Cognitive interviewing

#### Setting and participants

Participants were purposefully sampled from three nursing homes in South and East China. Participants who (1) provided direct care to people with dementia, (2) were aged 18 years or older, and provided informed consent were recruited in this study. The cognitive interviews were conducted as an iterative procedure. The sample size was determined based on information power^14^ and the guidance provided by the POS development team.^13^ An initial round of 5–10 individual interviews was conducted.^15^ These were analysed and informed revisions. A second round of 5–10 interviews was then conducted to test the revised IPOS-Dem.^15^

#### Data collection

The cognitive interviews were conducted via one-on-one interviews (virtually or in person). Participants were asked to think about a particular person with dementia with whom they are familiar and to whom they provide direct care. Any identifiable details of the person with dementia were not shared with the interviewer. All interviews were video/audio-recorded.

A topic guide informed by Tourangeau’s model of the survey response process was used to conduct the cognitive interviews. This model presents four cognitive steps involved in responding to surveys (comprehension, retrieval, judgement and response) and possible cognitive errors.^16^ Participants were asked about their thoughts on what was meant by each item and their response. Both the meaning of the items and responses were to be explored. The techniques ‘think aloud’ and verbal probing^17^ were used in the interview. In the ‘think aloud’ procedure, participants were asked to share their thinking as they were answering the questions. Concurrent verbal probing was used to elicit further information regarding the question interpretation and clarity.

#### Data analysis

The recordings were analysed using directed content analysis.^18^ A matrix developed from Tourangeau’s model^16^ in Microsoft Excel served to analyse individual interviews and then synthesise the results across all participants. Verbatim quotes were preserved throughout the analysis and synthesis and used to demonstrate the findings.

### Stakeholder consultation

A stakeholder group in China, comprising a person with dementia, two family members, a geriatric nurse, and a researcher, was actively engaged throughout the study. We consulted the group as a whole or individual members at different phases, which helped refine and modify items.

## Results

### Phase 1: Conceptual equivalence

Most terms within the IPOS-Dem are translatable. The terms ‘palliative care’ and ‘outcome measure’, as the fundamental concepts for understanding the scope of IPOS-Dem, offer several translation options. Three items had slightly different conceptual meanings based on different translations, including ‘Sore or dry mouth’, ‘Do you think s/he felt at peace?’ and ‘Has s/he been able to interact positively with others (e.g. staff, family, residents)’. The Chinese conceptual exploration of those concepts and items is listed in the Supplemental 1 Table S1.

### Phase 2: Forward translation

Two bilingual translators, a clinical physician and a linguist, independently completed two forward translations (FT1, FT2). There were grammatical and content differences in the two forward translations, regarding question phrasing, item terms and the Likert response categories.

There were some grammar and syntax differences in questions with regard to the length of sentence and request phrasing. The two forward translators suggested different wordings to explain three items: ‘Sore or dry mouth’, ‘Drowsiness (sleepiness)’ and ‘Wandering (as a result of distress or putting person at risk)’. And the two translators suggested different terms for the response endpoints ‘Not at all’ and ‘overwhelmingly’.

After discussion with the translators, these differences were resolved and agreed upon in FT3 (Supplemental 1 Table S2).

### Phase 3: Backward translation

Two bilingual translators, a clinical nurse and a humanities researcher, independently completed two backward translations (BT1, BT2). Both backward translations did not reveal significant discrepancies when compared to each other. There were five differences in the backward translation, including three items (‘Weakness or lack of energy’, ‘Drowsiness (sleepiness)’ and ‘Difficulty Communicating’) and two response categories (‘Overwhelmingly’ and ‘Cannot assess’).

### Phase 4: Expert review

There were seven main differences between the consensus version of backward translation (BT3) and initial English-language IPOS-Dem items. Main differences were identified among the five previously observed differences in the backward translations, along with an additional two items (‘Lost interest in things s/he would normally enjoy?’ and ‘Do you think s/he felt at peace?’). Supplemental 1 Table S3 presented the main results of backward translation and expert review. The prototype Chinese version was developed based on the discussion of the group with suggested wording revisions.

### Phase 5: Cognitive interviewing

#### Participants

Twelve participants from three Chinese nursing homes participated in two rounds of cognitive interviews, with six in each round. The nursing homes were located in urban areas across different regions of China, including Shanghai, Zhejiang and Fujian. Table 1 lists the demographic characteristics of participants in two rounds of cognitive interviews.

**Table 1.** Participant characteristics in two rounds of cognitive interviewing.

Characteristics	Round 1 (*N*)	Round 2 (*N*)
Age		
Median (min-max range)	37 (30–58)	42 (29–56)
Gender		
Female	5	5
Male	1	1
Education		
Master degree	2	1
Bachelor degree	2	2
Some college but no degree	2	2
High school degree	0	1
Professional background		
Nurse	4	3
Doctor/consultant	2	1
Healthcare assistant	0	1
Management	0	1
Experience in nursing homes (in years)		
0–3	1	3
4–6	2	2
7–9	3	0
10+	0	1

#### Item revision

Two rounds of interviews showed that most items and answer options worked well for the majority of participants. Reported difficulties were focused mainly on comprehension and a few concerned judgement and response. No problems were reported with retrieval. The challenges identified in the prototype version were resolved in the two rounds of cognitive interviewing.

The first round: The results of the first round of cognitive interviewing are presented under comprehension, retrieval, judgement and other concerns (Table 2). Difficulties were identified for comprehension (*n* = 8 items), judgement (*n* = 5 items), response (*n* = 4 items). Three other concerns were identified. Following discussions with the research team (CES, KES, AB), revisions were made ready for the second round of cognitive interviews (Table 2).

**Table 2.** Results from the first round of cognitive interviewing.

Corresponding step of Tourangeau’s model	Items (English and prototype Chinese version)	Problems	Quotations from interviewee(s)	Changes
Comprehension	Nausea (feeling like being sick/vomiting) 恶心 (感觉像是生病 了/感觉想呕吐）	All participants understood the meaning of ‘Nausea and vomiting’ The content in parentheses is not required and may cause confusion。	A002: ‘You can delete the word after nausea and vomiting, so that people will not make mistakes. In China, they must know about nausea and vomiting’.	Delete the content in parentheses.
	Vomiting (being sick) 呕吐 (生病了）			
	Drowsiness (sleepiness) 睡意 (睡眠）	Participants were not sure about (1) the exact meaning of drowsiness (sleepiness) and (2) how to distinguish drowsiness and sleep problems.	A002: ‘How do you separate drowsiness and sleep problems?’ ‘Drowsiness, does it often feel sleepy like this?’ ‘You can use some more obvious problems, such as feeling tired all the time’. A006: ‘Drowsiness is also serious. He sometimes wakes up occasionally, and his day and night are basically reversed’. ‘Is this a disease?’ ‘There is a bit of confusion with sleep problems, maybe it needs to be more detailed’.	(1) Use ‘昏昏欲睡(困倦) Drowsiness (lethargy)’ to translate ‘drowsiness (sleepiness)’ to see whether people can understand the exact meaning of the item. (2) Sleepiness means sleeping a lot during the day and night and then sleeping problems mean having trouble falling asleep, waking up at night, waking up early, etc.
	Difficulty communicating 沟通困难	Participants were not sure about what communication refers to. Some participants considered whether a resident with a different dialect causing communication difficulties should be considered when responding to this item.	A005: ‘Dialect, he couldn’t understand us, Can it be said that it was communication difficulty?’ A006: ‘Because he is not from our local area, and he has dementia. He is not deaf, and (he) often feels rebellious to what we say’.	Continue using ‘沟通困难’, as this is not a translation problem but with the scope of the concept of ‘communication’. Consider a broader definition of communication, including dialect, if it is causing distress.
	Sleeping problems 睡眠问题	Participants were not sure about (1) the exact meaning of ‘Sleeping problems’ and (2) how to distinguish drowsiness and sleep problems.	A003: ‘Sleep problems, do they refer to problems such as poor sleep, short sleep, and various other problems caused by using auxiliary methods or during the sleep process? But I can’t fully explain it’. A004: ‘The sleep problem is whether he gets enough sleep?’ A005: ‘(Compared to sleep problems) Sleep quality is better’. A005: ‘The quality of sleep depends on what time you go to bed, what time you wake up, how long a complete sleep lasts, what time you wake up in the middle, and how long you wait before falling asleep again’.	Use ‘sleeping quality problems’ (睡眠质量问题) instead of ‘sleeping problems’ (睡眠问题).
	Wandering (as a result of distress or putting person at risk) 游荡 (由于苦恼或使 人处于危险之中）	Participants did not comprehend this in the way that it was intended.	A004: ‘Does it mean sleepwalking?’ A006: ‘Does it mean that he makes others distressed? Putting person at risk, is it putting others in danger?’	Retranslate in the following order: Wandering (due to personal distress or putting oneself in danger). 游荡 (由于个人的苦恼或使自己处于危险之中)
	Lost interest in things s/he would normally enjoy? 他/她对日常感兴趣 的事情失去了兴趣 吗？	Participants were confused what ‘normally enjoy’ means.	A004: ’Because the things he liked when he was young are no longer applicable to him (it reminds me of his interests before he got sick). But does this refer to whether his recent interests are relatively stable?’	‘Has s/he lost interest in things s/he was recently interested in?’ 他/她对最近感兴趣的事情失去了兴趣吗？
	Do you think s/he felt at peace? 你认为他/她感到平静吗？	It is a challenge to translate ‘felt at peace’ in Chinese. Participants would like to know what ‘peace’ here refers to.	A003: ‘At peace, does this item mean that people with impaired consciousness like him can communicate normally or what?’ A003: ‘I can understand it after you explained it, but if you just look at this item, I don’t know what aspect you want to understand’. A004: ‘In terms of daily behaviour, if you are noisy or irritable, you are definitely not at peace. During this period, your behaviour is relatively stable, which means you are calm’. A006: ‘He doesn’t resist, at least he doesn’t show it’. A005: ‘. What exactly is he thinking in his heart? There may be some specific questions about what kind of peace he has’.	Use ‘peace and calmness’ (平静安稳) to see how people interpret that.
Judgement	Dental problems or problems with dentures 牙齿问题或假牙问题	Participants did not consider how an individual may be affected by dental, communication, sleeping, or wandering problems. Instead, they just described the problems that an individual may have. Participants felt there is a difference between ‘dental problems’ and ‘problems with dentures’. They were not sure about how to rate them in the same item.	A004: ‘His teeth just fell out. For some unknown reason, all he had left was the roots. Then how to rate here?’ A006: ‘He has no teeth anymore, they are all dentures, so why to rate? I don’t understand’. A004: ’How can we identify how many sleeping problems he has?’ A005: ‘Dialect, he couldn’t understand us, Can it be said that it was communication difficulty?’ A006: ‘How to judge the severity of wandering’.	An extra explanation was included at the beginning of IPOS-Dem. This was to emphasise the concept of ‘being affected by’ rather than the presence or absence of a symptom or concern. Respondents are asked to rate the degree to which individuals are affected by symptoms and concerns, rather than the presence and severity of a symptom or concern.
	Difficulty communicating 沟通困难			
	Sleeping problems 睡眠问题			
	Wandering (as a result of distress or putting person at risk) 游荡 (由于苦恼或使 人处于危险之中）			
	Do you think s/he felt at peace? 你认为他/她感到平静吗?	Participants were not sure about what information about the person to use when making the judgement for this item.	A005: ‘What do you want to recall about him in terms of ‘at peace? Is it more about whether he was calm?’ A002: ‘This is so difficult to evaluate, especially for Chinese people. What they say is different from what they think in their hearts, and sometimes they can’t tell the difference. He acts peacefully in front of you, but his real behaviour is totally different. It’s too difficult to assess ‘at peace’ with assessments’.	Use ‘peace and calmness’ (平静安稳)
Response	Difficulty communicating 沟通困难	Participants demonstrated difficulty in rating the severity of these items.	A005: ‘Do you have a standard for this? What situation is worth 0 points and what is 1 point? This will be more reference for scoring. If there is no specific standard, when the same patient will be evaluated differently by different people, the score is different’.	An additional explanation was added to help respondents understand the meaning of the response categories and how to interpret the results.
	Wandering (as a result of distress or putting person at risk) 游荡(由于苦恼或使 人处于危险之中)		A001: ‘How to quantify this 01234? How to rate it as 1 or 3? There are more subjective factors here’.	
	Do you think s/he felt at peace? 你认为他/她感到平静吗？		A005: ‘How to evaluate using degree rating, because this is a subjective part, you can’t tell it from the appearance’.	
	Cannot assess 无法评估	Some participants were not able to understand the meaning of ‘cannot assess’ response option.	A006: ‘It seems that ‘cannot assess’ is not easy to understand. ‘Cannot assess this item/no problem identified’ seems better.’	Use ‘cannot assess this item’. (无法评估该条目)
Other identified problems	Title 痴呆症综合安宁疗护结果量表	There are multiple terms used for dementia in China. The most common name in Chinese is used in the policy documents but some researchers and clinicians argue that it may have a ‘discriminatory tone’ and can easily make people with dementia feel stigmatised. However, the alternative term is less commonly used and may be less well understood.	A001: ‘It seems that some people also use ‘认知症’ or ‘认知障碍’ right? But it sounds too formal and academic, and many ordinary people might not understand it’. A002: ‘I think ‘失智症’ is better and sounds more acceptable’ A006: ‘I think it’s good to call it ‘失智症’, but (compared to dementia) everyone knows better about ‘痴呆症’, which means memory loss and the inability to take care of oneself. Maybe these can be understood’.	Use the more positive name and give the reason in the explanation page. Revised title: 失智症综合安宁疗护结果量表
	Please write clearly Person’s name Person’s number Date (dd/mm/yyyy) 请清楚填写 患者姓名 患者号码 日期(日/月/年)	Participants did not understand what number is required here.	A003: ‘When people move in, you can call them patients. If it’s a number, is it the bed number?’ A006: ‘The number is not easy to understand. Are you asking his hospital number, or his contact number? His mobile phone number, or what is it? I think it could be clearer’.	Ensuring that the number can help respondents identify the individual with dementia.
	Who was involved in this assessment? Please tick all that apply: Person with dementia Keyworker Care team Family member(s) Health professional Other Please state 谁参与了这次评估？请勾选适用项 痴呆症患者 关键人员 护理团队 家庭成员 卫生专业人员 其他请说明	Participants did not understand the meaning of keyworker. Participants did not know the difference between care team and health professional.	A004: ‘Who are the keyworker? I feel like these are key people’ A006: ‘Who is involved in this assessment? Am I a team member? Who are the keyworkers? Care workers?’	Who was involved in this assessment? Please tick all that apply: Person with dementia Family member(s) Care team Doctor Nurse Care staff/assistant Other Please state 谁参与了这次评估？请勾选适用项 失智症患者 家庭成员 医疗照护团队 医生 护士 卫生辅助人员/护理员 其他请说明

The second round: Most revisions made based on the results of the first round resolved identified problems. Four items were identified with comprehension difficulties. Additionally, one other difficulty was noted. Subsequent to discussions with the research team (CES, KES, AB), revisions were made. Table 3 provides a detailed overview of the interview results and the subsequent modifications made based on them.

**Table 3.** Results from the second round of cognitive interviewing.

Corresponding step of Tourangeau’s model	Items (Original items and revised version after first cognitive interviewing)	Problems	Quotations from interviewee(s)	Revision
Comprehension	Original: Drowsiness (sleepiness) 睡意(睡眠) Revised: Drowsiness (lethargy) 昏昏欲睡(困倦)	Participants were not able to understand ‘昏昏欲睡(困倦) Drowsiness (lethargy).	B001: ‘Want to sleep but can’t?’ ‘If ‘Drowsiness (sleepiness)’ means sleeping a lot during the day and nights, the ‘somnolence (嗜睡)’ in Chinese would fit’. B005: ‘Being drowsy means being in a state of not being quite awake all the time’. B006: ‘Drowsiness and lethargy are somewhat similar. Some people don’t really want to sleep, but their mental state is not good. The two expressions are very similar. Drowsiness means wanting to sleep, being sleepy, and sometimes lethargy also refers to the limbs’.	Use ‘somnolence’(嗜睡) to reflect the intention of this item (excessive daytime and night-time sleepiness). Add the definitions for ‘somnolence’(嗜睡) and ‘sleeping quality problems’ (睡眠质量问题) in the explanation page.
	Original (no revision): Difficulty communicating 沟通困难	When broader definition of ‘communication’ was adopted, people were interpreting it into different meanings.	B001: ‘Due to the dialect problem, we couldn’t understand what the old man said’. B004: ‘Unable to express things accurately. . .unable to fully express what he means’. B005: ‘He may not be able to convey what he means, and the two parties may be communicating ineffectively’. B006: ‘Communication difficulties may be more about the kind of unclear communication within yourself, rather than that you don’t understand what I’m saying’.	Add the following definition in the explanation page: Adopt a broader definition of communication, which can include all factors that affect communication, such as decreased expression ability (due to dementia or other diseases), dialect problems, and inaccurate wording (due to education and life background).
	Original: Do you think s/he felt at peace? 你认为他/她感到平静吗? Revised item: Do you think s/he felt peace and calmness 你认为他/她感到平静安稳吗?	Participants did not consider spirituality. Two participants suggested using ‘inner peace’, to better reflect the component of spirituality.	B003: ‘Emotions, can he calm down for a moment or a period of time? Is being in a daze considered calm? What I may understand is “daze” and “mind goes blank”. . .For us, it may mean calming down emotionally and behaviorally. . .“physical silence”’. B005: ‘Calm? Why is it called calmness (安稳)?’ ‘When talking about deep peace, inner peace seems easier to understand rather than this (calmness)’.	Use ‘inner peace’ (内心平静) to come close implicitly to religion or spiritual concerns in Chinese culture.
	Original (no revision): Have all practical problems been addressed? [e.g. hearing aids, foot care, glasses, diet] 是否所有实际问题都得到了解决？ (例如助听器, 足部护理, 眼镜, 饮食)	Participants felt the term ‘problems’ seemed very vague and suggested the term ‘needs’.	B006: ‘Have all the practical needs being met? I think it is better. Because the ‘practical problem’ feels very general and vague.	Have all practical problems and needs been addressed? [e.g. hearing aids, foot care, glasses, diet] 是否所有实际问题和需求都得到了解决？ (例如助听器, 足部 护理, 眼镜, 饮食)
Other identified problems	Original (no revision): Please write clearly Person’s name Person’s number Date (dd/mm/yyyy) 请清楚填写 患者姓名 患者号码 日期(日/月/年)	Participants suggested making it clear that the number here is the number that can identify the resident. Participants suggested deleting ‘person/patient’ before the terms ‘name’ and ‘number’ and using ‘yyyy/mm/dd’ rather than ‘dd/mm/yyyy’.	B001: ‘Just name and number, or call them old people’ B006: ‘Number? telephone number? Sick? For clinical patients, there may be more bed numbers’.	Add the explanation about the identification number and present the content in the following way. Please write clearly Name Number (A number that can identify the resident) Date (yyyy/mm/dd) 请清楚填写 姓名 号码 (可识别该居民的号码） 日期 (年/月/日）
Additional comments	B005: ‘The explanation on the first page is needed and helpful to explain the focus of the scale. It is better to have this explanation, otherwise sometimes there is ambiguity’. B006: ‘You may need to provide certain explanations or training with the person using IPOS-Dem. Group training or video training could also be considered’.			

### Stakeholder consultation and final version

After the cognitive interviewing (phase 5) was completed, the National Health Commission of the People’s Republic of China released the naming regulation for commonly used clinical medical terms, in which the standard naming of ‘palliative care’ was reidentified. The whole stakeholder group was consulted about the choice of language and final version. The research team decided to adopt the new terminology in line with regulations and some wording changes. The final IPOS-Dem Chinese version was approved by POS development team. Table 4 shows the comparison between the English-language IPOS-Dem and the final culturally adapted Chinese IPOS-Dem version. The Chinese version is freely available at https://pos-pal.org/.

**Table 4.** Original English and final culturally adapted Chinese versions of IPOS-Dem – all items.

Initial IPOS-Dem item	Chinese IPOS-Dem items (Bold indicates changes were needed after discussion to agree on a final translation)
Title: Integrated Palliative Outcome Scale for People with Dementia	Title: Integrated Palliative Outcome Scale for People with Dementia **失智症综合缓和医疗结果量表 (IPOS-Dem)**
Please write clearly Person’s name Person’s number Date (dd/mm/yyyy)	Please write clearly **Name** **Number (A number that can identify the person)** **Date (yyyy/mm/dd**) **请清楚填写:** **姓名** **号码 (可识别此人的号码):** **日期(年/月/日)**
Pain	Pain **疼痛**
Shortness of breath	Shortness of breath 呼吸短促
Weakness or lack of energy	Weakness or lack of energy 虚弱或乏力
Nausea (feeling like being sick/vomiting)	**Nausea** **恶心**
Vomiting (being sick)	**Vomiting** **呕吐**
Poor appetite	Poor appetite 食欲不振
Constipation	Constipation 便秘
Dental problems or problems with dentures	Dental problems or problems with dentures 牙齿问题或假牙问题
Sore or dry mouth	Sore or dry mouth 口疮或口干
**Drowsiness (sleepiness)**	**Somnolence** **嗜睡**
Poor mobility (trouble walking, cannot leave bed, falling)	Poor mobility (trouble walking, cannot leave bed, falling) 行动不便 (行走困难, 不能下床, 跌倒)
Swallowing problems (e.g. chokes, inhales food or drink, holds food in mouth)	Swallowing problems (e.g. chokes, inhales food or drink, holds food in mouth) 吞咽问题 (例如噎食, 误吸食物或水, 将食物含在嘴里）
Skin breakdown (redness, skin tearing, pressure damage)	Skin breakdown (redness, skin tearing, pressure damage) 皮肤破损 (发红, 皮肤破溃, 压力性损伤)
Difficulty Communicating	Difficulty Communicating 沟通困难
Sleeping problems	**Sleeping quality problems** **睡眠质量问题**
Diarrhoea	Diarrhoea 腹泻
Hallucinations (seeing or hearing things not present) and/or delusions (fixed false beliefs)	**Hallucinations and/or delusions** **(Hallucinations: seeing or hearing things not present. Delusions: fixed false beliefs.)** **幻觉和/或妄想** **(幻觉：看到或听到不存在的东西。妄想：固定的错误信念。）**
Agitation (restless, irritable)	Agitation (restless, irritable) 躁动 (不安, 烦躁）
Wandering (as a result of distress or putting person at risk)	**Wandering (due to personal distress or putting oneself in danger)** **游荡 (由于个人的苦恼所致或使自己处于危险之中)**
Has s/he been feeling anxious or worried?	Has s/he been feeling anxious or worried? 他/她是否感到焦虑或担心？
Have any of his/her family or those important to them, been anxious or worried about the person?	**Have any of his/her family or those important to them, been anxious or worried about the person?** **他/她的家人或重要的人是否为他/她感到焦虑或担心？**
Do you think s/he felt depressed?	Do you think s/he felt depressed? 你认为他/她感到沮丧吗？
Lost interest in things s/he would normally enjoy?	**Has s/he lost interest in things s/he was recently interested in?** **他/她最近是否对感兴趣的事情失去了兴趣？**
Do you think s/he felt at peace?	**Do you think s/he felt inner peace?** **你认为他/她感到内心平静吗？**
Has s/he been able to interact positively with others (e.g. staff, family, residents)?	Has s/he been able to interact positively with others (e.g. staff, family, residents)? 他/她是否能够与他人积极地互动 (例如工作人员, 家人, 其他居民）
Have all practical problems been addressed? [e.g. hearing aids, foot care, glasses, diet]	**Have all practical problems and needs been addressed? [e.g. hearing aids, foot care, glasses, diet]** **是否所有实际问题和需求都得到了解决？ (例如助听器**, **足部护理**, **眼镜**, **饮食）**
Who was involved in this assessment? Please tick all that apply Person with dementia Keyworker Care team Family member(s) Health professional Other Please state	Who was involved in this assessment? Please tick all that apply: **Person with dementia** **Family member(s)** **Care team** **Doctor** **Nurse** **Care staff/assistant** **Other Please state** **谁参与了这次评估？请勾选适用项** **失智症人士** **家庭成员** **医疗照护团队** **医生** **护士** **卫生辅助人员/护理员** **其他 请说明**

Other changes: • Add an extra explanation page: To explain the reason for using the more positive name to translate Dementia, the focus of this measure (the concept of ‘being affected’), how to interpret the result and the meanings of some items. • Add an explanation of the meaning of ‘Difficulty Communicating’ in the explanation page: Adopting a broader definition of communication, which can include all factors that affect communication, such as decreased expression ability (due to dementia or other diseases), dialect problems and inaccurate wording (due to education and life background). • Add an explanation of the meaning of ‘Somnolence’ and ‘Sleeping quality problem’ in the explanation page to distinguish these two items.

## Discussion

### Summary

This study describes the translation and cultural adaptation of IPOS-Dem into Chinese, marking the first holistic outcome measure for people with dementia in China. Rigorous translation and cultural adaptation process could enable the IPOS-Dem Chinese version to be equivalent to the original. While most items were conceptually aligned, certain items posed challenges in comprehension and judgement. Additionally, careful consideration was required in naming the measure in Chinese.

### Comprehension challenges

Several distinct differences in comprehension were found in items ‘Do you think s/he felt at peace?’ and ‘Difficulty communicating’.

The term ‘at peace’ generated most of the controversy in the cognitive interviews. The term was debated for its meaning, which was similar to other IPOS translation groups.^19–21^ People often associated calmness in emotion and behaviour with the term ‘peace’, missing the spiritual aspect of peace. However, in the Chinese context, it was necessary to exercise discretion when addressing spiritual matters. Therefore, based on the insights gathered from participants, we used ‘inner peace (内心平静)’ to come close implicitly to religion or spiritual concerns in Chinese culture.^22,23^

The item ‘Difficulty communicating’ was unequivocal in the translation process. However, during cognitive interviewing, individuals interpreted it diversely, setting it apart from other IPOS-Dem translation groups.^5,11,24,25^ Since any cause of communication problems may lead to distress, a broader definition of communication was adopted in the Chinese version to reflect the holistic nature of IPOS-Dem, which can include all factors that affect communication, such as decreased expression ability (due to dementia or other diseases), dialect problems and inaccurate wording (due to education and life background).

### Judgement challenges

Judgement challenges surfaced predominantly in how to comprehend and apply the concept of ‘being affected’ when answering the items. The presence and severity of the symptoms and concerns were first considered, while the subjective burden experienced tended to be overlooked when participants made judgements. It implies that person-centredness, the cornerstone of palliative care, is not yet well-grounded, similar to findings of other translation groups in other cultural contexts.^5,19^ An additional concern arose regarding how proxy reporting reflects person-centeredness, specifically in gauging whether proxy reporters accurately convey individuals’ perspectives on their health. This aspect could be further explored through psychometric testing, including inter-rater reliability testing. Drawing from the experiences of another IPOS translation groups, both patient self-report and staff proxy-report versions of IPOS have been approved to be valid and reliable.^9^ Nonetheless, the agreement between patient and proxy reports varies across different contexts.^9,26,27^

We found that training and education could be pivotal in overcoming the judgement challenges that became apparent from cognitive interviews, in line with the findings of German IPOS-Dem translation group.^5^ Acquiring a solid understanding of palliative care, person-centred care and outcome measures would shape users’ perspectives on the measures themselves. Moreover, a shared understanding of the purpose and significance of the measure, along with practical training on how to use, report and interpret IPOS-Dem would support the successful application of IPOS-Dem in the clinical setting and help reduce subjectivity.^5,28–30^

### Measure naming

Palliative care is a core term in the name of the measure. China has previously used ‘安宁疗护’ as an inclusive term to collectively describe hospice care, terminal care, palliative care and other terms related to palliative care.^31,32^ However, in March 2024, the National Health Commission of the People’s Republic of China released the naming regulation for commonly used clinical medical terms,^33^ in which ‘安宁疗护’ is used to describe ‘hospice care’ and ‘缓和医疗’ is used to describe palliative care. As such, we consulted with five stakeholders and IPOS-Dem development team. Given that the importance of adhering to policy guidance and the long-term timeliness of future use, we decided to use the redefined term ‘缓和医疗’ as the name for the measure.

Translating the term ‘outcome’ presents a significant challenge. Currently, most Chinese literature uses ‘结局’ as the translation.^34–36^ However, in English ‘outcome’ refers to a ‘change in current or future health status attributable to a preceding healthcare intervention’.^37^ The term ‘结局’ does not fully capture the concept of ‘change’ inherent in ‘outcome’, as it typically denotes the final or last stage in a story’s plot or situation.^38^ Thus, we have chosen to use ‘结果’ as the translation, which better conveys both the notion of a phenomenon arising from other phenomena and the final state of development and change at a certain stage.^39^

Another critical term in naming the measure is dementia. There are several Chinese translations for this term. The most commonly used name and most widely understood, ‘痴呆症’ in Chinese, was perceived to have a ‘discriminatory tone’ and easily made people with dementia feel stigmatised.^40^ The participants suggested using the less common term ‘失智症’ to diminish stigmatisation. We embraced this suggestion with the aim of reducing stigma, aligning with the perspective of some Chinese researchers.^41,42^ However, we acknowledge that not everyone may be familiar with the new term and further explanation is required. In addition, the chosen name may not be perfect in completely achieving de-stigmatisation. De-stigmatisation is a gradual process, during which the terminology may evolve to aid in stigma reduction.

The naming of the measure reflects the context and cultural considerations, shedding light on the understanding of palliative care and dementia care within the current context. We propose that naming the measure should not be seen as a definitive endpoint determined by a single decision, but rather as a dynamic process that acknowledges and embraces cultural and contextual changes.

### Strengths and limitations

The study followed the precise methodology set by the POS development team for cross-cultural adaptation. Stakeholders became collaborative partners in the study. They played a significant role in guiding decisions to ensure the study’s outcomes were more likely to benefit patients and the wider community. We purposefully selected participants from three nursing homes located in different cities to enhance geographical and care experience diversity. Although the Chinese IPOS-Dem was tested with nursing home staff, it is likely to be relevant in broader settings. The relatively small sample size in the cognitive interviewing phase needs to be acknowledged. Nevertheless, it’s worth noting that this sample size was aligned with the guidance provided by the POS development team^13^ and wider research.^21,43–45^ Given that cognitive interviewing yields rich data, it’s not uncommon to have small sample sizes in such studies.^46–48^

## Conclusion

The IPOS-Dem was translated and culturally adapted for Chinese. This study proved essential in refining the translation of IPOS-Dem to ensure the interpretation of and potential responses to each item by Chinese users in Chinese contexts reflect the original English IPOS-Dem item’s meaning. This study presents the first person-centred outcome measure for the substantial population of individuals with dementia in China, offering the potential for improved care and outcomes for this population. Education and training are required to improve the use of IPOS-Dem.

## Supplemental Material

sj-docx-1-pmj-10.1177_02692163251347826 – Supplemental material for Translation and cross-cultural adaptation of Integrated Palliative Care Outcome Scale for Dementia Supplemental material, sj-docx-1-pmj-10.1177_02692163251347826 for Translation and cross-cultural adaptation of Integrated Palliative Care Outcome Scale for Dementia by Linghui Chen, Katherine E Sleeman, Huichan Huang, Yihan Mo, Andy Bradshaw and Clare Ellis-Smith in Palliative Medicine
