# Supplementary material for: Translation and cross-cultural adaptation of Integrated Palliative Care Outcome Scale for Dementia
Source: Palliat Med. 2025 Jul 13;39(8):897–909. doi: 10.1177/02692163251347826 (PMC12405654; doi:10.1177/02692163251347826)
Supplement: sj-docx-1-pmj-10.1177_02692163251347826 – Supplemental material for Translation and cross-cultural adaptation of Integrated Palliative Care Outcome Scale for Dementia [file sj-docx-1-pmj-10.1177_02692163251347826.docx]

**Supplementary 1**

| **Table S1. Conceptual definition exploration** | |
| --- | --- |
| Key concepts | Conceptual definition exploration in Chinese context |
| Palliative care | - Palliative care improves the quality of life of patients and that of their families who are facing challenges associated with life-threatening illness, whether physical, psychological, social or spiritual. The quality of life of caregivers improves as well.   *(Who Health Organization:* [*https://www.who.int/news-room/fact-sheets/detail/palliative-care*](https://www.who.int/news-room/fact-sheets/detail/palliative-care)*)*   - In previous policy documents, China didn’t strictly distinguishes hospice care, terminal care, palliative care and other terms related to palliative care, but clearly refers to hospice care, terminal care, end of life care, palliative care etc. collectively as ‘安宁疗护’.   *(赵越, 刘兰秋. 英国和美国社区居家安宁疗护服务模式及其对我国的启示 [J] . 中国全科医学, 2022, 25(19) : 2330-2335. DOI: 10.12114/j.issn.1007-9572.2022.0256.)*   - Chinese definition for ‘安宁疗护’：中国将临终关怀、舒缓医疗、姑息治疗等统称为安宁疗护，是指为疾病终末期或老年患者在临终前提供身体、心理、精神等方面的照料和人文关怀等服务，控制痛苦和不适症状，提高生命质量，帮助患者舒适、安详、有尊严地离世。   *(中华人民共和国国家健康卫生委员会: National Health Commission of the People’s Republic of China http://www.nhc.gov.cn/wjw/tia/201801/162783d72e26439ea4d0d670e76d79bf.shtml)* |
| Outcome measure | - The term "outcome measure" can be translated into Chinese in several word-for-word ways. The term ‘结果’, ‘结局’ or ‘产物’ which means ‘results’, ‘consequence’ or ‘output’, are more lay Chinese expressions for ‘outcome’. The term ‘measure’ could be understood as ‘测量’, ‘评估’ or ‘评价’. - Based on the Chinese literature, the definition of ‘**结局/结果测量**’ might be appropriate. The Chinese definition could be: 结局测量是评价与量化患者结局的工具。   *(陈千吉,陈红,张英, 等. (2021). 患者报告结局测量工具选择路径：以中国腰痛患者日常生活活动能力量表的选择为例. 中国全科医学 (36), 4648-4652+4660.)*   - Donabedian defined an outcome as a ‘**change** in current or future health status attributable to a preceding healthcare intervention’^1^. A change in health status in the palliative care population is, for instance, an improvement or a worsening of a symptom (e.g., pain or breathlessness)^2^. Within palliative care, measuring outcomes is important because they allow us to evaluate whether the care that is given to patients and their families makes a difference to their quality of life^3^.  1. Donabedian A. Explorations in quality assessment and monitoring (Vol. I, 1980; Vol. II, 1982; Vol. III, 1985). Ann Arbor, MI: Health Administration Press 1980. 2. de Wolf-Linder S, Dawkins M, Wicks F, et al. Which outcome domains are important in palliative care and when? An international expert consensus workshop, using the nominal group technique. Palliative Medicine 2019; 33: 1058-1068. DOI: 10.1177/0269216319854154. 3. Dudgeon D. The impact of measuring patient-reported outcome measures on quality of and access to palliative care. Journal of palliative medicine 2018; 21: S-76-S-80.   There are two different translations for ‘outcome’. To embrace the concept of ‘change’ within the definition of ‘outcome’, it is better to adopt ‘**结果**’ as the translation.  1. 结果 /jiéguǒ/   - 在一定阶段事物发展变化的最后状态。The final state of a thing's development and change at a certain stage. - 哲学上指由他事物或现象而产生的事物或现象（跟「原因」相区别）。In philosophy, it refers to a thing or phenomenon that arises from other things or phenomena (as opposed to "cause").   2. 结局 /jiéjú/   - 最终的局面；故事情节发展的最后阶段。The final situation; the last stage in the development of a story's plot. |
| Sore or dry  mouth | The exact translation for ‘sore’ would be ‘痛’, ‘疮’or ‘溃疡’. However previous experience and tools had shown that people may understand better the term ‘疮’ which would be translated as ‘ulcer’ in English. |
| Do you  think s/he felt at peace? | The terms ‘at peace’ were not identified in most literature. It has many options for translating ‘peace’, such as ‘平静’, ‘平安’, ‘和睦’, ‘和平’, ‘安乐’, ‘静穆/穆’, ‘泰然’。 |
| Has s/he been  able to interact  positively with others (e.g. staff,  family, residents) | The term ‘interact’ could be understood as ‘mutual/reciprocal action or influence’. However, it could be translated as ‘互动’, ‘互相作用’, or ‘互相影响’. To close the intention of initial IPOS-Dem, ‘互动’ might be the appropriate word. |

| **Table S2. Forward translation dissimilarities and consensus** | | | | |
| --- | --- | --- | --- | --- |
| Initial item | FT1 | FT2 | FT3 (consensus) | Rationale |
| Question: Please select one box that best describes how the person has been affected by each of the following symptoms over the past week. | 请选择一个最能描述此人在过去一周内如何受到以下各种症状影响的方框。 | 请选择一个方框，该方框能最好地描述过去一周内患者受到以下每种症状的影响的程度 | 请选择一个方框，该方框能最好地描述过去一周内患者受到以下症状影响的程度。 | We chose translation in FT2 because by splitting the sentences one can better understand what the boxes represent. |
| Question: Has the person had any other symptoms? Please select one box to show how you feel each of these symptoms have affected the person over the past week (optional). | 有任何其他症状吗？请选择一个方框来说明您认为这些症状如何在过去一周内影响此人（可选）。 | 在过去的一周，是否有其他症状影响他，并选择相应的方框（可选）。 | 有任何其他症状吗? 请选择一个方框来说明您认为在过去一周，每个症状如何在影响此人(可选)。 | We chose to make some modifications to the translation in FT1 because this translation best reflects the original format of the question. |
| Sore or dry mouth | 口痛或口干 | 口干、咽痛 | 口疮或口干 | FTI and FT2 differ in the translation of ‘sore’. We decided to use ‘疮’ to be consistent with the experience with previous measures. |
| Drowsiness (sleepiness) | 困倦（瞌睡） | 想睡（入睡困难） | 睡意(睡眠) | FT1 and FT2 had different understandings of this item, resulting in different translations. We decided to use the most literal translation ‘睡意(睡眠)’. |
| Wandering (as a result of distress or putting person at risk) | 游荡（遇险或使人处于危险之中） | 梦游（使人置于危险中） | 游荡(由于困扰或使人处于危险之中) | We chose to use the translation from FT1 with modifications. Because FT2 mistakenly believes that this may refer to sleepwalking. |
| Response endpoint: Not at all | 无 | 一点也没有 | 一点也没有 | ‘一点也没有’ better reflets the original format and meaning of this response endpoint. |
| Response endpoint: overwhelmingly | 非常严重 | 超出承受范围 | 超出承受范围 | ‘超出承受范围’ better reflects the degree of overload. |

| **Table S3. Results from backward translation (BT) and expert review (ER)** | | | | | | |
| --- | --- | --- | --- | --- | --- | --- |
| Initial English-language item | FT3 item | BT1 | BT2 | BT3 (consensus) | Prototype version |  |
| Weakness or lack of energy | 虚弱或乏力 (Identified from BT, ER) | Weakness or fatigue | Tiredness or fatigue | Weakness or fatigue | 虚弱或乏力 |  |
| Drowsiness (sleepiness) | 睡意(睡眠)  (Identified from BT, ER) | Sleepiness(sleep) | Drowsiness (sleep) | Drowsiness (sleep) | 睡意（睡眠） |  |
| Difficulty Communicating | 沟通困难  (Identified from BT, ER) | Communication deficit | Difficulty speaking | Communication deficit | 沟通困难 |  |
| Lost interest in things s/he would normally enjoy? | 他/她对以往日常感兴趣的事情失去了兴趣吗?  (Identified from ER) | Has he/her lost interest in those he/her used to be interested in in daily life? | Did he or she lose interest in things that he or she used to be interested in every day? | Did he or she lose interest in things that he or she used to be interested in every day? | 他/她对日常感兴趣的事情失去了兴趣吗？ |  |
| Do you think s/he felt at peace? | 你认为他/她感到平静吗?  (Identified from ER) | Do you think he/her feels calm? | Did he/she appear to be calm? | Do you think he/her feels calm | 你认为他/她感到平静吗？ |  |
| Overwhelmingly | 超出承受范围地  (Identified from BT, ER) | Unbearable | Intolerable | Intolerable/Unbearable | 超出承受范围地 |  |
| Cannot assess | 无法评估  (Identified from BT, ER) | Immeasurable | Inaccessible | Inaccessible/Immeasurable | 无法评估 |  |
